# Role of Microbial Modulation in Management of Atopic Dermatitis in Children

**DOI:** 10.3390/nu9080854

**Published:** 2017-08-09

**Authors:** Lies Hulshof, Belinda van’t Land, Aline B. Sprikkelman, Johan Garssen

**Affiliations:** 1 Department of Paediatric Respiratory Medicine and Allergy, Emma Children’s Hospital Academic Medical Center, 1105 AZ Amsterdam, The Netherlands; l.hulshof@amc.uva.nl; 2 Department of Paediatric Immunology, University Medical Center Utrecht/Wilhelmina Children’s Hospital, 3584 EA Utrecht, The Netherlands; b.vantland@umcutrecht.nl; 3 Nutricia Research, 3584 CT Utrecht, The Netherlands; 4 Department of Paediatric Pulmonology and Paediatric Allergology, University Medical Center Groningen, 9713 GZ Groningen, The Netherlands; a.b.sprikkelman@umcg.nl; 5 Utrecht Institute for Pharmaceutical Sciences, Faculty of Science, Utrecht University, 3584 CG Utrecht, The Netherlands

**Keywords:** atopic dermatitis, children, mucosal immune development

## Abstract

The pathophysiology of atopic dermatitis (AD) is multifactorial and is a complex interrelationship between skin barrier, genetic predisposition, immunologic development, skin microbiome, environmental, nutritional, pharmacological, and psychological factors. Several microbial modulations of the intestinal microbiome with pre- and/or probiotics have been used in AD management, with different clinical out-come (both positive, as well as null findings). This review provides an overview of the clinical evidence from trials in children from 2008 to 2017, aiming to evaluate the effect of dietary interventions with pre- and/or pro-biotics for the treatment of AD. By searching the PUBMED/MEDLINE, EMBADE, and COCHRANE databases 14 clinical studies were selected and included within this review. Data extraction was independently conducted by two authors. The primary outcome was an improvement in the clinical score of AD severity. Changes of serum immunological markers and/or gastrointestinal symptoms were explored if available. In these studies some dietary interventions with pre- and/or pro-biotics were beneficial compared to control diets in the management of AD in children, next to treatment with emollients, and/or local corticosteroids. However, heterogeneity between studies was high, making it clear that focused clinical randomized controlled trials are needed to understand the potential role and underlying mechanism of dietary interventions in children with AD.

## 1. Introduction

Worldwide, the most common inflammatory skin disease is atopic dermatitis (AD) with a prevalence of 10–20% in children [1]. In 60% of these children, the onset of AD occurs early in life, before one year of age [2]. Pediatric AD can be characterized by its relapsing-remitting nature and the overall severity is mild in most of these young children [3]. The pathophysiology is multifactorial with a complex interrelationship between skin barrier development, genetic predisposition, immunological development, skin microbiome composition, and environmental, nutritional, pharmacological, and psychological factors. Whether or not AD is a primarily driven barrier dysfunction or a primarily inflammatory skin disease remains open for debate. Taking into account the recent understanding of the complex role of host microbial development in early life, new insights on regarding the role of microbial modulation in AD development during infancy may be hypothesized.

### 1.1. Host–Microbiome Development and Nutrition

The first contact of mucosal tissues to external microbiota is crucial in the establishment and maturation of the mucosal, as well as systemic, immune systems [4,5]. In particular, the first year of life is essential for programming the immune system. The development of barrier function and the immune system are influenced by environmental factors, such as feeding patterns, antibiotic use by the mother during delivery, or postnatal use of antibiotics by the neonate [6]. Proper understanding of the protective and programming effects of a healthy immune and microbiome development may provide opportunities to reduce the risk of development of AD. Any discordance between the early developmental requirements of the infant’s immune system may contribute to the development of allergic diseases [7]. A recent COCHRANE systematic review of five clinical trials (952 participants) concluded that avoiding major allergens in the maternal diet (during gestation/lactation) does not protect against development of AD in the infant during the first 18 months of life [8]. This study also concluded that there was insufficient evidence that prolonged exclusive breast feeding was protective against AD. Early sensitization to food allergens through breast milk, skin contact, and/or inhalation occurs and may explain why some infants show an allergic response to specific proteins despite having never ingested it [7]. An additional influencing factor in allergy development is the timing of solid food introduction. For instance, infants starting with solid food introduction at four or five months of age had a lower risk for AD development (Odds ratio = 0.41, 95% Confidence interval, 0.20–0.87) compared to infants which were exclusively breastfed [9]. However, except maybe for peanut allergy, strong evidence is lacking to decide whether the age of complementary food introduction should be four or six months in order to prevent the development of allergy [5]. Although scientific evidence is limited, it has been suggested that timing of the start and type of nutrition (i.e., breastfeeding/infant formula) during solid food introduction influences the development of allergic diseases [10]. The question, however, remains whether observed effects are derived from direct interaction with immune cells, or indirectly through alterations in the microbiome composition and change in derivatives thereof, followed by immune changes [11]. The microbial composition is involved in the development of the regulatory T cell response and thereby plays a key role in immune development [12]. Within in vitro assays it has been shown that the addition of specific oligosaccharides during dendritic cell development induces a regulatory T cell response potentially of benefit in an allergic setting [13]. Moreover, dietary supplementation with specific prebiotic oligosaccharides has been shown to reduce the risk of developing allergies in infants [14]. Therefore, during early life it seems likely that specific components can contribute to the normal immune development via multiple direct and indirect pathways, thereby reducing the risk of allergic manifestations.

### 1.2. Development of Skin and Microbiome in Early Life

The composition and diversity of the skin microbiome shows a unique habitat per location, especially within children. The skin microbiome composition may be affected by different factors including age, sex, and microbial antigen exposure. Skin microbiota of neonates varies by the mode of delivery, but the differences become less apparent with age in early childhood [15,16]. The composition is also dependent on pH, temperature, Ultraviolet (UV) exposure, natural moisturizing factors (NMFs), and can easily change over time [17]. Specific changes in the skin microbiome have been associated with AD and other allergic manifestations [18]. AD has been associated with early life colonization of *Staphylococcus aureus* (*S. aureus*). A reduced bacterial diversity in the skin microbiome is of major importance in AD pathogenesis [19]. Only 5% of the skin microbiome in non-atopic individuals is colonized with *S. aureus*, compared to 39% in non-lesional skin and 70% in lesional skin of AD patients [20,21]. Although within a birth cohort it was shown that 10 infants with AD at the age of 12 months were not colonized with *S. aureus* before their first AD manifestation [22], colonization and infection with *S. aureus* has been associated with increased IgE responses, food allergy, and severity of AD skin disease [23,24]. In addition to the bacterial composition, the fungal and viral community differences are also associated with allergic manifestations such as rhinitis and asthma, as well [25,26]. This underscores the complexity of the host-microbe balance induction, as well as the sensitivity towards modulations herein [27].

Although whether or not AD is primarily driven by microbial dysbiosis leading to barrier dysfunction remains a key question, the skin barrier is hampered in AD. Within infants the skin has a higher ability to restore itself as a barrier. This adaptive flexibility results in unique properties of infant skin [28]. In a recent meta-analysis of genome-wide association studies of more than 15 million genetic variants in 21,399 cases and 95,464 controls, 10 new loci associated with AD risk were identified, bringing the total susceptibility loci until to 31 at the time of this publication [28,29,30]. Children with AD have changes in their skin barrier due to filaggrin deficiency or tight-junction dysfunction, which allows the penetration of irritants, allergens, and bacteria, leading to inflammation [31,32].

### 1.3. Immune Deregulation within AD

In AD, the skin barrier function is compromised, allowing penetration of environmental factors, such as irritants, allergens, and bacteria, leading to inflammation and/or allergic sensitization [33,34]. Skin barrier dysfunction induces the release of several inflammatory factors, including thymic stromal lymphopoietin (TSLP) and other cytokines and chemokines, which trigger inflammation in the skin [35]. These pro-inflammatory mediators (including chemokines) are released by the affected keratinocytes to attract leukocytes to the site of inflammation [36,37]. The characteristic leukocyte migration into the skin is driven by excessive chemokine production at the site of inflammation. Several chemokines (classically characterized within the Th1-type of response: CXCL9, CXCL10, and CXCL11; the Th2-type response: CCL-17, CCL22; and for inflammation: CCL-20) have been associated with an AD phenotype comprising complex pathology [38]. Studies on the pathology of early paediatric AD are limited and correlation of disease activity has been shown with only a few serum biomarkers (i.e., CCL17, CCL22, CCL27, and IgE) in infants [38,39]. Recently, profound immune activation in non-lesional skin in paediatric patients with AD has also been detected [38]. While little is known about the alterations in skin-derived immunity and skin barrier function that occur during the early-onset phase of AD, Th2 (IL-13, IL-31, and CCL17), Th22 (IL-22 and S100As), and some Th1-skewing (IFN-γ and CXCL10) have been detected in the skin, which is also observed in adults [38]. An increase in recruited Th2 cell populations classically leads to the increased production of interleukins IL-4, IL-5, and IL-13, which may be locally involved in the induction of IgE and eosinophil activation [39]. However, the identification of the trigger in AD development is still very complex.

### 1.4. Current Understanding in the Specific Microbial Modulations in AD

Due to the potential role of the microbiome in children with AD and development in early childhood, this seems a promising time frame for effective nutritional interventions including those with pre- and/or probiotics. In 2008 a comprehensive COCHRANE review (analysing 10 clinical trials (up to April 2008 (781 children)) concluded that overall probiotics in general seem not to be effective as a treatment of AD [40]. On the contrary, a modest role for probiotic interventions in paediatric dermatitis was suggested [41,42], as well as within later studies regarding pre- and/or probiotic interventions [43]. As stated by the recent meta-analysis by Kim et al. [44], the difference between age and severity of AD as measured by scoring of childhood atopic dermatitis (SCORAD) should be taken into account when analysing the impact of microbial modulations. Collectively, there was a large heterogeneity between trials complicating the comparison between specific species, strains, dosage, duration, time or age, and clinical outcomes. The aim of this review is to give an overview of the results of recently performed clinical intervention studies published after the COCHRANE review in 2008 until June 2017, studying the effect of microbial modulations for treatment of AD in children.

## 2. Methods

### 2.1. Search Strategy

To identify clinical dietary intervention studies with prebiotics/probiotics and/or synbiotics in children with AD, from birth up to 18 years of age, the PUBMED/MEDLINE, EMBASE, and COCHRANE databases have been searched. Since the COCHRANE review included all clinical trials up to April 2008, the literature search started from 2008 to June 2017. The following keywords were used: (probiotics OR prebiotics OR synbiotics) AND (atopic dermatitis OR eczema).

### 2.2. Study Selection

Published clinical intervention studies were included within this review meeting the following criteria: clinical studies with a dietary intervention with prebiotic(s) and/or probiotic(s), all participants were human, more specifically children from birth up to 18 years of age, all children with AD before the start of intervention, only studies with a clinical outcome for AD severity, studies published in the last 10 years, written in English, and presenting original data. A total of 75 abstracts have been retrieved through the database searches. Two authors independently checked the fulfilment of the inclusion criteria for this review by screening titles and abstracts and excluded studies that obviously did not fulfil the inclusion criteria. Seventy-three were published between April 2008 and 2017. A total of 59 from the 73 studies were excluded for this review, due to different outcomes than the inclusion criteria for this overview. The majority of these clinical studies had prevention of AD or other allergic manifestations as the primary outcome (N = 31). Other reasons to exclude studies were: no clinical AD outcome value, only gastrointestinal outcomes, safety studies, or genetic outcomes. In addition, three long-term follow-up studies were excluded. Finally the reference lists of the positively identified articles were checked for additional clinical studies and led to one additional study.

### 2.3. Data Extraction

Data extraction was independently conducted by two authors and cross-checked to avoid errors. Disagreements were resolved through consensus and, when needed, using the opinion of a third author. Details of the study were recorded: methods, objectives, study population, age of children with AD, inclusion and exclusion criteria, mild-to moderate-to severe AD, mean SCORAD scores, specific dietary intervention, strain and dosage of prebiotics and or probiotics, control diet, duration of intervention, number of randomized children in each group, clinical outcome of AD severity and change in AD severity (using primarily the AD scoring system SCORAD [45]). In addition, if available, the immunological outcomes, as well as the gastrointestinal outcomes, were included.

## 3. Results

### 3.1. Study Characteristics

The literature search resulted in 75 clinical intervention studies and, after inclusion, 13 studies could be used. One publication included two different types of interventions; open label versus randomized control using the same probiotic strain. Therefore, those two clinical studies will be mentioned separately in this overview. The study characteristics of the 14 selected clinical trials (N = 1008 children with AD) are summarized in Table 1 [43,46,47,48,49,50,51,52,53,54,55,56,57]. 

### 3.2. Specific Dietary Intervention

Since the results of clinical intervention studies in the past showed inconclusive results of prebiotics and/or probiotics in the treatment of AD, it was hypothesized that the beneficial effects of prebiotics and probiotics are strain- and dose-dependent. *Lactobacillus* and *Bifidobacterium* are the two most investigated bacterial species in allergy research. Again, a wide variety in the dietary intervention studies was found in the clinical trials between 2008 and 2017 (Table 1). In total, 12 included trials were randomized controlled trials (RCTs) which had a total of 601 AD children in the treatment groups and 444 AD children in the control groups. The two additional clinical trials provided an open label intervention and were conducted with 63 children with AD. Not one intervention study used the same strain of probiotics or the same combination with prebiotics. More specifically, five RCTs provided synbiotic mixtures [43,47,49,50,55], four RCTs provided only one probiotic as dietary intervention [52,54,56,57], two RCTs provided more than one probiotic strain within the study [48,53], one trial explored the effect of prebiotics only [46], and the two open label studies were conducted with only one probiotic strain [51,57].

### 3.3. Effect of Dietary Intervention on Clinically-Detected AD Severity

In total, 12 out of the 14 studies reported the severity in AD using SCORAD. Three of the five RCTs with synbiotic intervention showed significant AD improvement by reduction of AD severity after dietary intervention compared to control diet [49,50,55]. The remaining two synbiotic RCTs showed a significant reduction of SCORAD score in both groups [43,47]. Improvement of AD severity was shown in three out of the four RCTs with one probiotic strain [52,54,57]. In addition, the result of the two open label intervention studies with one strain of probiotics is an improvement in AD symptoms [51,57]. Additionally, the RCT with the highest enrolment of children with AD (N = 220), showed an improvement in AD severity compared to the control group [53]. The trial of Gore et al. with two strains of probiotics showed a reduction of the SCORAD score in both groups [48]. The prebiotic trial showed improvement of AD severity in both groups [46]. In addition, the RCT with synbiotic intervention showed improvement of AD severity (SCORAD score). In the trial of van der Aa et al., a subgroup analysis of AD infants with elevated IgE levels showed a greater SCORAD score reduction in the synbiotic group [43]. A previous study also showed that synbiotic interventions may have beneficial effects, especially in AD with elevated IgE [58]. Taking IgE further into account, Shafiei et al. found no differences in comparing SCORAD scores in the IgE and non-IgE AD infants [47]. In contrast to Wang et al., who included only AD children with at least one positive skin prick test or at least one elevated specific IgE level within their study, showed improvement in AD [53], suggesting an important role for IgE.

### 3.4. Additional Effect of Dietary Interventions

Knowing the complexity of development in early life, some studies provide additional exploratory markers within the different RCTs. Three RCTs showed changing levels in cytokines IL-4 and/or IFN-y, which were significantly lower after intervention or did not show any change [51,52,53]. Additionally, changes in serum chemokine markers were detected, showing significant changes in two [54,57] groups, and no significant differences between groups in another study [43]. Some studies showed changes in IgE levels, but no significance between treatment and control [43,48,53]. In addition to serum markers, a few studies provide additional data on the effect of dietary intervention cellular composition [50]. For instance, the eosinophil count was significantly lower between groups in one study [52], and over time in one other study [55]. In three studies no immunological data was available, which may be due to the young age of infants included in those studies [46,47,51]. Only three RCTs reported on faecal bacterial counts after dietary intervention; all, however, confirmed changes in gut microbial composition after dietary intervention. Gastrointestinal symptoms, such as stool consistency and diaper dermatitis, were also positively influenced in the treatment group compared to control after the specific dietary interventions [43,46].

### 3.5. Treatment Duration and Time of Intervention

Within the identified clinical intervention studies, the treatment duration was variable, ranging from eight weeks up to six months [43,46,47,48,49,50,51,52,53,54,55,56,57]. There seemed to be no association between the duration of dietary intervention and the outcome of clinical improvement of AD. The trials indicating improvement of AD in both groups had intervention periods of eight or 12 weeks [43,46,48]. The effective duration of dietary intervention remains unclear. In two trials included in this review, a positive beneficial effect on AD severity remained detectable after the intervention period [53,55].

## 4. Discussion

This review summarizes the clinical outcome on AD severity among children receiving dietary intervention with or without prebiotics, probiotics, or synbiotics. It presents an overview of data from 2008 to June 2017 regarding the use of the specific dietary interventions in the treatment of AD in children, from birth up to 18 years of age available to date. As in earlier studies and meta-analyses published up to 2008, overall, strong evidence to clarify insights into the role of pre-, pro-, and synbiotics in treatment of AD is lacking, which may be due to high heterogeneity among the trials. Moreover, the outcome on AD severity seems to depend of multiple factors, including age, season, UV exposure, the use of local corticosteroids, number of AD exacerbations, etc.

Within the clinical intervention studies included in this review, several confounding factors need to be considered when interpreting the results. For example, since it is unethical to withhold AD children AD treatment with emollients and/or topical steroids, most studies provided treatment simultaneously with the dietary intervention. However, not all trials reported the amount and frequency of topical steroid use and, therefore, it is not clear if children in the treatment group were using the same amount of topical steroids as in the control groups. Within future studies, the method of randomisation, as well as amount and frequency of used topical steroids, should be reported. Although in some trials a decrease of local steroid use was mentioned, the effect of nutritional intervention compared to pharmacological treatment may both influence the AD severity score. For clinical outcome comparisons, the SCORAD score is validated for AD severity, but it is known to be difficult to assess in young infants, providing possible inter-observer variability. Moreover, due to the relapsing-remitting nature of AD in these young children (which occurs randomly in time), this may complicate the evaluation of AD severity and the impact of nutritional intervention effects in time. Recently, the evaluation on AD measurements provided recommendations about scoring AD severity, providing an interesting tool for future research. These include SIS (skin intensity score) (paediatric version), POEM (patient-oriented eczema measure), SCORAD (Severity Scoring of Atopic Dermatitis index), SA-EASI (self-administered eczema area and severity index score), and adapted SA-EASI, which are currently the most appropriate instruments to assess AD and, therefore, should be recommended as core symptom instruments in future clinical trials [59].

With the knowledge that both the child’s microbiome and mucosal immunity evolves from infancy into early childhood, the age of inclusion in a study of AD is of utmost importance. In infants the cheeks are a typical preferred site of AD and the nasal tip is usually spared. After one year of age, a change of AD sites is more prone to the antecubital and popliteal fossa. Possible explanation for this change is change in the diversity in the skin microbiome and changes in skin barrier function in early life. Nevertheless the age of inclusion was within the dietary intervention studies, which may explain the differing results. Hypothesizing that the first year of life is the essential period for programming the mucosal immune system, this may also be the preferred time to prevent the development of AD with microbial modulations. However, as shown within some recent meta-analyses with a specific focus on the clinical evidence from dietary intervention studies, it was concluded that dietary intervention during pregnancy, or lactation in the mothers, or in the early life of the infant led to a decreased risk of AD development, but not the development of other allergies [60,61,62]. It should be noted that the overall evidence is low due to the inconsistent results among studies. In order to investigate and understand the mechanisms involved in immune modulation capacities of microbiota by dietary interventions on clinical outcome severity of AD in childhood, the consistency between trials must increase. Therefore, criteria should be formulated on how to conduct future studies in this field in order to be able to compare clinical trial outcomes, so that subsequently reliable and valid advice can be given to implement dietary interventions in the management of AD. 

## 5. Conclusions

Identifying high-risk children for atopic manifestations and AD children who can benefit from these microbial modulations seems highly relevant. Moreover, a better understanding of the contributing factors, such as the skin microbiome, faecal composition, and biomarkers of skin barrier function, leading to changes within serum biomarker profiles would be of great value for a more individualized therapeutic approach in AD management in children. In addition, to overcome the problem of heterogeneity of the studies and therefore the limitation of comparing clinical trial outcomes, an international committee of experts should focus on definitions of outcome measures, treatment duration, administration of the product, etc. Since the development and evolution of each child’s microbiome starts from infancy up to childhood, an early life dietary intervention (in pregnancy and/or in infancy) seems preferable. Further standardized clinical research is, however, necessary to gain more insight into specific strains, prebiotics and timing for optimal intervention. By combining the individual factors of a child with AD, as mentioned above, it may be possible to eventually match the clinical needs with the best dietary management option available.

## Figures and Tables

**Table 1:** Effects of microbial modulation in children with AD.

Subjects (Age, N, Treatment vs. Control)	Inclusion Criteria	Dietary Intervention	Treatment Period and Dose of Pre/Probiotics	Primary Parameter	Clinical Outcome, AD Severity and IgE	Immunological Outcomes	Gastro Intestinal Outcomes	Reference
Term infants 6–8 weeks N = 120 60 vs. 60	Positive history of allergy in one parent or sibling No breastfeeding at inclusion	Hydrolysed formula (HA) with GOS Control diet Only HA formula	For six months; Per 100 mL GOS 0.5 g	Differences in SCORAD score	After dietary intervention decrease of SCORAD in both groups (ns)	No serum data was available	Significant softer stool consistency in prebiotic group (*p* < 0.05)	Bozensky, et al. 2015 [46]
Term infants 0–7 months N = 89 42/47	SCORAD > 15 No more breastfeeding at inclusion No antibiotics four weeks before inclusion	Extensively hydrolysed formula with *Bifidobacterium Breve* M-16V and GOS/FOS Control diet Only extensively hydrolysed formula	For 12 weeks; Per 100 mL *BB.* 1.3 × 10^9^ CFU GOS 0.72 g (90%) FOS 0.08 g (10%)	Change in severity of AD	After dietary intervention significant reduction of SCORAD in both groups. In subgroup of 50 infants, with elevated IgE levels, improvement in SCORAD after 12 weeks was greater in synbiotic group compared to control diet (*p* = 0.04)	No differences in spec IgE after 12 weeks between groups, No significant differences on IL-5, IgG1, IgG4, CCL17 and CCL27 after 12 weeks between groups. Significant increase of total IgE levels in both groups	Faecal pH was significantly lower in synbiotic group (*p* = 0.001) Significant softer stool consistency in synbiotic group (*p* = 0.05). Diaper dermatitis less prevalent in synbiotic group (*p* = 0.008)	Van der Aa, et al. 2010 [43]
Infants 1–36 months N = 36 18/18	Moderate to severe AD (SCORAD > 25)	Daily sachet with 7 strains of probiotics and FOS Control diet Daily sachet 1000 mg sucrose	For eight weeks; 10 mg probiotic mixture of 1 × 10^9^ CFU 990 mg FOS	Clinical effect	After dietary intervention the mean total SCORAD in both groups decreased by 56% of all children. No differences between groups. In IgE + subgroup, similar decrease of AD severity in both groups	No serum data was available	No gastro intestinal data was available	Shafiei, et al. 2011 [47]
>34 weeks gestation 3–6 months N = 137 90 vs. 47	SCORAD>10 >200 mL standard formula daily	Extensively hydrolysed formula with a sachet *Lactobacillus paracasei CNCM I-2116* or with a sachet *Bifidobacterium lactis CNCM I-3446* Control diet; Extensively hydrolysed formula with maltodextrin sachet	For 12 weeks; *LP.* 10^10^ CFU *BL.* 10^10^ CFU *LP*. N = 45 *BL*. N = 45 C. N = 47	Change in SCORAD	After dietary intervention SCORAD reduction decreased significantly over time in all groups	No significant effect of probiotic treatments on the prevalence of allergen sensitization post-intervention	No differences in infants administered the L/M-permeability test between the groups	Gore, et al. 2012 [48]
Infants 3–72 months N = 40 19 vs. 21	Mild to severe AD No prior exposure to antibiotics or probiotics	1 g sachet with a mixture of 7 probiotic strains and FOS Control diet; 1 g sachet with placebo powder	For eight weeks; Twice daily mixture of 1 × 10^9^ CFU and FOS	Change in AD severity	After dietary intervention greater reduction in SCORAD in synbiotic group compared to control diet (*p* = 0.005)	No significant differences on cytokine production of IFN-y or IL-4 between groups	No gastro intestinal data was available	Farid, et al. 2011 [49]
Infants 12–36 months N = 90 43 vs. 47	Moderate to severe AD (8 vs. 9 children with proven DBPCFC milk or egg allergy two months before inclusion on a milk or egg diet)	*Lactobacillus achidophilus DDS-1* and *Bifidobacterium lactis UABLA-12* and FOS in a rice maltodextrin powder Control diet; Pure powder of rice maltodextrin	For eight weeks; Twice daily Mixture of *LA.* and *BL2.* 5 × 10^9^ CFU and 50mg FOS	Percentage change in SCORAD	After dietary intervention greater decrease in mean SCORAD in synbiotic group compared to control diet (*p* = 0.001)	Absolut count of CD4 and CD25 lymphocyte subsets were decreased whereas CD8 count increased in synbiotic group after dietary intervention compared to control diet.	No gastro intestinal data was available	Gerasimov, et al. 2010 [50]
Children 0–11 years N = 43	AD symptoms	Sachet *Lactobacillus salivarius LS01* *DSM22775* No control diet	For eight weeks; Twice daily *LS*. 1 × 10^9^ CFU	Change in AD severity	After dietary intervention significant reduction SCORAD in N = 28 (*p* = 0.001)	No serum data was available	No gastro intestinal data was available	Niccoli A, et al. 2014 [51]
Children 1–13 years N = 83 44 vs. 39	SCORAD ranged from 20 to 50	*Lactobacillus plantarum CJLP133* Control diet; placebo preparation No fermented food products containing live microorganisms were allowed	For 12 weeks; Twice daily *LP2.* 0.5 × 10^10^ CFU	Improvement of clinical and immunological parameters in children with AD	After dietary intervention greater decrease in SCORAD compared to control (*p* = 0.004)	Total eosinophil counts, Logarithmic IFN-y and IL-4 were significantly lower after dietary intervention in probiotic group compared to control (*p* = 0.023) (*p* < 0.001) (*p* = 0.049)	No gastro intestinal data was available	Han, et al. 2012 [52]
Children 1–18 years N = 220 165 vs. 55	AD symptoms > 6 months before inclusion SCORAD >15 At least 1 positive SPT or spec. IgE antibodies to common allergens	Capsule with *Lactobacillus para-casei GMNL-133* or capsule with *Lactobacillus fermentum GM090* or capsule with both probiotics Control diet; Placebo capsule	For three months; *LP3*. 2 × 10^9^ CFU *LF*. 2 × 10^9^ CFU *LP3 + LF* 4 × 10^9^ CFU *LP3.* N = 55 *LF.* N = 53 *LP3 + LF* N = 51 C. N = 53	Change in AD severity	After dietary intervention (three groups LP, LF, and LP + LF mixture) lower SCORAD compared to control (*p* < 0.001) Difference remained at four months after discontinuing the probiotics	Total IgE levels were reduced within the *LP* and *LP + LF* group, but no significant differences compared to control. Significant change in IL-4 compared to control (*p* = 0.04)	The probiotics groups had significant higher fecal colony counts of *Bifidobacterium* (*p* = 0.004) and lower counts of *Clostridium* (*p* = 0.03) compared to control	Wang IJ, et al. 2015 [53]
Children 2–10 years N = 75 41 vs. 34	AEDS for six months prior to study Total SCORAD > 25	Microcrystalline cellulose with *Lactobacillus sakei KCTC 10755BP* Control diet; only microcrystalline cellulose	For 12 weeks; Twice daily *LS2.* 5 × 10^9^ CFU	Evaluation of clinical outcome of AD	After dietary intervention mean change in Total SCORAD was significantly greater in probiotic group compared to the control group (*p* = 0.008)	Serum CCL17 and CCL27 levels were significantly decreased in probiotic group compared to control (both *p* < 0.001)	No gastro intestinal data was available	Woo, et al. 2010 [54]
Children 2–14 years N = 54 27 vs. 27	AD symptoms for at least four days SCORAD > 25	Capsule with *Lactobacillus salivarius PM-A0006* and FOS Control diet; Capsule with corn starch and FOS	For eight weeks; Twice daily 25 mg *LS3*. (2 × 10^9^ CFU), 475 mg FOS Control; 25 mg corn starch 475 mg FOS	SCORAD changes	After dietary intervention SCORAD significant lower in synbiotic group compared to prebiotic group (*p* = 0.022), differences remained at week 10	The median serum eosinophil cationic protein decreased significant within the groups but not significant different between the groups	No gastro intestinal data was available	Wu, et al. 2012 [55]
Children 4–10 years N = 51 26 vs. 25	AD symptoms No antibiotics for eight weeks No local corticosteroid use for eight weeks prior to study	Chewable tablet with *Lactobacillus reuteri ATCC55730* Control diet; chewable placebo tablet	For eight weeks; Once daily *LR.* 1 × 10^8^ CFU	Effects on exhaled breath condensate (EBC) cytokine expression	After dietary intervention, no significant changes SCORAD mean values in probiotic group compared to control group	EBC IFN-y increased and IL4 decreased significantly in 16 IgE positive AD children in probiotic group compared to 14 IgE positive AD children in the control group (both *p* = 0.001)	No gastro intestinal data was available	Miniello, et al. 2010 [56]
Children 4–15 years N = 20	AD No cow’s milk spec IgE	Fermented milk with *Lactobacillus* *acidophilus L-92* No control diet	For eight weeks; once daily 150 mL milk + *LA2*. 3 × 10^10^ CFU	Symptom-medication score (SMS), which is calculated as sum ADASI and calculated medication score of less topical steroid use.	Changes in ADASI, in SMS, and itch (all three; *p* < 0.001)	No changes in blood biochemical parameters, including the total plasma IgE concentration.	Significant decrease in the total faecal Bacteroidaceae count (*p* = 0.034), Significant increase in the faecal Lactobacillus count (*p* = 0.007)	Torii S, et al. 2010 [57]
Children 1–12 years N = 50	AD No cow’s milk spec IgE	Fermented milk with dried and heat-killed *Lactobacillus acidophilus 92* and dextrin. Control diet; Fermented milk with dextrin	For eight weeks; once daily 150 mL milk + heat-killed *LA2* 1.5 × 10 ^11^ CFU + 900 mg dextrin	Symptom-medication score (SMS).	Significantly decreased of SMS in probiotic group compared to control group (*p* = 0.0127)	Changes in CCL17 levels were significantly different between probiotic group compared to control group (*p* < 0.01)	No gastro intestinal data was available	Torii S, et al. 2010 [57]

The clinical dietary intervention studies have been ordered according to the age of the children at inclusion. Abbreviations Table 1: AD, atopic dermatitis; IgE, Immunoglobulin E; HA, hydrolysed infant formula; GOS, galacto-oligosaccharides; SCORAD, scoring atopic dermatitis score; FOS, Fructo-oligosaccharides; BB, *Bifidobacterium breve M-16V*; CFU, colony-forming units; LP, *Lactobacillus paracasei CNCMI-2116*; BL *Bifidobacterium lactis CNCMI-3446*; C, control group; DBPCFC, double blind placebo controlled food challenge; LA, *Lactobacillus achidophilus DDS-1*, BL2 *Bifidobacterium lactis UABLA-12*; LS *Lactobacillus salivarius LS01 DSM22775*; LP2, *Lactobacillus plantarum CJLP133*; LP3, *Lactobacillus para-casei GMNL-133*; LF, *Lactobacillus fermentum GM090*; LS2 *Lactobacillus sakei KCTC1075*5*BP*; LS3*, Lactobacillus salivarius PM-A0006*; LR, *Lactobacillus reuteri ATCC55730*; LA2, *Lactobacillus acidophilus L-92.*

## Abbreviations

AD	Atopic dermatitis
CCL	CC chemokine ligand (−17, −20, −22, −27)
CXCL	CXC chemokine ligand (−9,−10,−11)
EASI	Eczema area and severity index
FLG	Filaggrin
lcFOS	long chain—Fructo-oligosaccharides
scGOS	short chain—Galacto-oligosaccharides
IgE	Immunoglobulin E (total, specific)
IL	Interleukin (−4, −5, −13, −22, −31)
IFN	Interferon (−γ)
NMF	Natural moisturizing factors
POEM	Patient-oriented eczema measure
RCT	Randomized controlled trial
SA-EASI	Self-administered eczema area and severity index score
SASSAD	Six Area, Six Sign Atopic Dermatitis severity score
SIS	Skin intensity score
SCORAD	Scoring atopic dermatitis score, clinical tool for scoring AD severity
SC	Stratum corneum
TEWL	Trans epidermal water loss
Th-	T helper cell type (1, 2, 17, 22)
TIS	Three Item Severity score
TJ	Tight junction
TSLP	Thymic stromal lymphopoietin
UV	Ultraviolet
